# Viewing garden scenes: Interaction between Gaze Behavior and Physiological Responses

**DOI:** 10.16910/jemr.13.1.6

**Published:** 2020-05-13

**Authors:** Congcong Liu, Karl Herrup, Seiko Goto, Bertram E. Shi

**Affiliations:** HKUST Clear Water Bay, Hong Kong; Nagasaki University, Japan; University of Pittsburgh, USA

**Keywords:** Eye movement, Markov models, Attention, Physiological responses, Interaction, Gender differences, Eye tracking, Region of interest

## Abstract

Previous research has shown that exposure to Japanese gardens reduces physiological measures of stress, e.g. heart rate, in both healthy subjects and dementia patients. However, the correlation between subjects’ physiological responses and their visual behavior while viewing the garden has not yet been investigated. To address this, we developed a system to collect simultaneous measurements of eye gaze and three physiological indicators of autonomic nervous system activity: electrocardiogram, blood volume pulse, and galvanic skin response. We recorded healthy subjects’ physiological/behavioral responses when they viewed two environments (an empty courtyard and a Japanese garden) in two ways (directly or as a projected 2D photograph). Similar to past work, we found that differences in subject’s physiological responses to the two environments when viewed directly, but not as a photograph. We also found differences in their behavioral responses. We quantified subject’s behavioral responses using several gaze metrics commonly considered to be measures of engagement of focus: average fixation duration, saccade amplitude, spatial entropy and gaze transition entropy. We found decrease in gaze transition entropy, the only metric that accounts for both the spatial and temporal properties of gaze, to have a weak positive correlation with decrease in heart rate. This suggests a relationship between engagement/focus and relaxation. Finally, we found gender differences: females’ gaze patterns were more spatially distributed and had higher transition entropy than males.

## Introduction

Non-pharmacological interventions, such asphysical activity programs, cognitive intervention, sensory intervention, behavioral therapy, and environmental modification, areincreasingly attractinginterest for the management of Alzheimer disease and dementia(1). These are often associated with fewer adverse side effects, e.g. sedation, abnormal gait, extrapyramidal symptoms, and even accelerated cognitive decline, than pharmacological interventions are.

A popular form of environmental modification is the integration of healing gardens, which are increasingly being deployed in healthcare facilities (2).Healing gardens are designed to provide a safe and secure therapeutic environment, with elements such as wide continuous paths that provide direct exposure to nature.Friedrich et al. suggest that garden exposure can help address memory deficits, reduce anxiety and agitation in dementia patients (3). Pasha et al. suggest that healing gardens have valuable therapeutic benefits for hospital patients (4). Revee et al. found that a healing garden in a children's hospital provides a sense of calm and peacefulness (5).

Anumber of theories have been proposed to explain the beneficial health effects of natural landscapes.Kaplan and collaborators developed the Attention Restorative Theory, whichproposes that the involuntary attention evoked by natural environments counteracts on the mental fatigue arising from the sustained voluntary attention required by daily work(6).Ulrich et al. forwarded the Stress Recovery Theory, which predicts that natural scenes encourage recovery, whereas urban settings hamper recovery from stress (7).

Certain garden designs seem be more effective than others in eliciting these responses. Japanese gardens, which are designed to be calming (8), may be more effective than others.Goto et al. quantified the physiological and psychological responses of Japanese viewers exposed to three different landscapes: a Japanese tea garden, a French garden and forest space, using heart rate and self-reports to the Profile of Mood States (POMS) (9). Exposure to the tea garden was associated with the greatest reductions in heart rate and had the greatest relaxing effect.Similar experiments conducted with healthy elderly Caucasian subjects also showed that a Japanese garden elicited the largest beneficial responses compared to two other spaces (10).Elsadek et al.compared the effects of viewing three different gardens: a landscape garden, a Japanese garden and an architectural garden, on university students from Japan and Canada. Exposure to the Japanese garden led to more fixations and thehighest scores for good atmosphere and garden design(11).Dementia patients responded positively when exposed to a Japanese garden, but negatively when exposed to a Snoezelen room (12). Viewing a Japanese garden reduced heart rate and improved behavioral symptoms of Japanese subjects with dementia, more than viewing a control space did(13).

As described by (14), there are several design principles that may lead to differences in gaze behavior when subjects view Japanese versus western style gardens. The style of the Japanese garden developed over hundreds of years to help practitioners of meditation achieve their desired mental state. The design of Japanese gardens encourages viewers to slow down their gaze and to engage with the natural elements.

Japanese gardens are naturalistic while western gardens tend to be geometric. The design of the Japanese garden is an allusion to nature. It encourages the viewer to imagine a larger landscape from a small, seemingly simple natural element. There are many ways to create this illusion, such as composing rock, pruning trees and not placing sculptures in the garden.

A geometric garden is designed to lead the eye directly toward a single focal point, emphasizing a single point perspective composition by the garden elements. Although Japanese gardens do contain a main object, there are other sub-elements placed in the front or on the side, whichlead the viewer to see the main object via the sub-elements. The sub-elements include trees, which partially screen the view of the main object, or rocks, which point the viewer's attention towards the main object. Thus, a Japanese garden has multiple focal points to induce multi-directional viewing. The principle is that if the viewer looks too directly at the main object, it is very boring.

While the design principles and the calming effects of Japanese gardens are well established, the ways in which viewers interact visually with the garden, and how this interaction might lead to the calming effects of Japanese gardens has not been well studied. To address this issue, we developed a measurement system enabling us to track a subject's gaze non-intrusively and to record their physiological signalssimultaneously (15, 16, 17).The physiological signals measured are the electrocardiogram, the blood volume pulse and the galvanic skin response.To our knowledge, this is the first system enabling simultaneous monitoring subjects' visual interaction with a real 3D environment and their physiological responses.

We used this system to record the behavioral and physiological responses of healthy subjects when viewing two spaces (a Japanese garden and a control space) in two ways(directly and as a projected photograph).

The four main contributions of the work are as follows. First, we report on the first set of synchronized measurements of eye gaze and physiological responsesas subjects view garden scenes. While previous work has measured eye gaze and physiological responses during the same session, these measurements were not synchronized, which made the analysis of precise correlations as we perform here impossible. Second, we find evidence that the spatiotemporal dynamics of subjects’ eye movements differ in different scenes, and that these dynamics correlate with their physiological responses.Third, our results add further support to past work showing gender differences in visual behavior. Although gender differences in exploratory eye movements has been studied with very different stimuli or experimental designs (18, 19, 20), to our knowledge no previous studies have examined gender differences in physiological and behavioral responses to garden viewing. Consistent with the past studies mentioned above we find that females engage in more exploratory behavior than males. Fourth, we find differences in the visual behavior between viewing the garden directly or as a photograph.Subjects' gaze was more evenly distributed over the visual elements when viewing the photograph, but more concentrated on particular elements when viewing the scene directly.

## Methods

This section describes the experimental setup for collecting measurements, the experimental protocol followed, and the methods used to analyze the gaze and physiological data.

### Experimental setup

**Figure 1.**
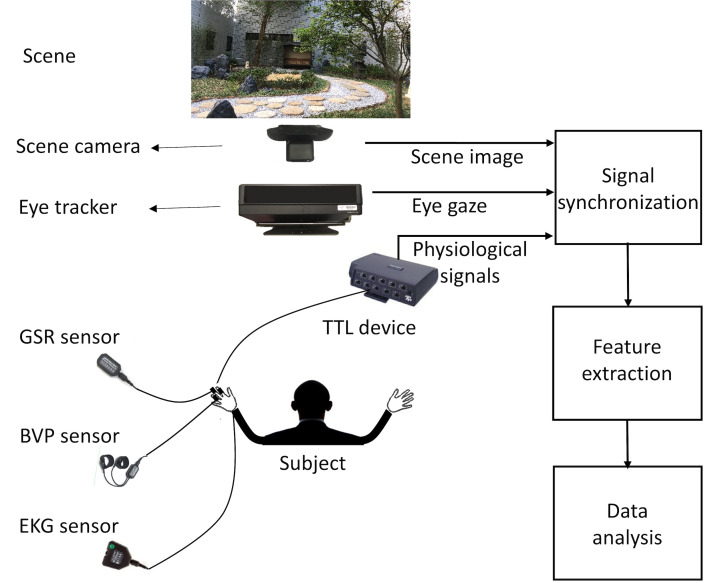
System diagram of our experiment setup. The TTL device is a ProComp Infiniti system by Thought Technology Ltd. GSR denotes galvanic skin responses. BVP denotes blood volume pulse. EKG denotes electrocardiogram.

Figure 1 shows the main components of the system we developed to collect simultaneous measurements of eye gaze and physiological responses. 

Eye gaze data is captured at 60 Hz with a Tobii Pro X60 eye tracker. The scene camera, a Logitech C920 camera, captures a 960 x 720 pixel image of the scene. Measurements of eye gaze in eye tracker coordinates were projected to points in the scene camera image using the algorithm described in (15).Both the eye tracker and the scene camera are remote from the user, making the eye gaze measurements non-intrusive so as to interfere with subject's natural gaze behavior as little as possible. 

Physiological data is collected by a ProComp Infiniti system usingthree sensors attached to one hand of the subject. We collected the electrocardiogram (EKG) using a wrist sensor with a sampling frequency of 2048 Hz, the blood volume pulse (BVP) using fingertip sensor mounted on the ring finger with a sampling frequency of 256 Hz, and the galvanic skin response (GSR) using a pair of electrodes attached to the index and middle finger with a sampling frequency of 256 Hz.

We synchronized the eye gaze and physiological measurements by attaching an optical sensor to the ProComp Infiniti. This sensor measured the infrared illumination generated by the eye tracker for each eye gaze measurement.

**Figure 2.**
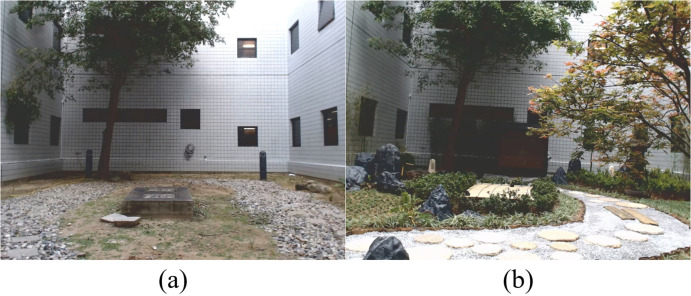
Pictures of the two visual environments the subjects viewed: (a) a simple courtyard with one tree, (b) a Japanese garden.

Figure 2 (a) and (b) shows photographs of the two scenes viewed by the participants. Both scenes were of the same physical space (16.5m x 10.1m area), but landscaped in two different ways. This ensured that most conditions for the two scenes were the same, except for the visual content. 

In the first landscape design, most elements were removed from the space, leaving a simple courtyard with only a large tree, a water hose, black lamp posts, some gravel, and a concrete block in the middle of the space. The second landscape design was a Japanese garden consisting of many more visual elements. Common elements between the two scenes were the large tree and the concrete block. The black lamp posts and gravel were removed. The water hose was obscured by a Japanese bench. New elements included an Acer Palmatun maple tree, large rocks, a gravel pathway, a Japanese kasuga stone lantern, Azalea shrubbery, a water element and the Japanese style bench.The visual appearance of the concrete block was altered by a Japanese tatami map covering its top surface, and the shrubbery obscuring the sides of the block.

Photograph viewing experiments were conducted in an indoor windowless office. Lights were turned off during viewing. The photographs shown in Figure 2 were projected onto a 120cm high and 160cm wide area using an LCD projector (EB-925, EPSON, 3500 lumens, XGA 1024 x 768).The physical dimensions of the projection were chosen so that the same visual element spanned the same visual area in the direct and projected photograph viewing sessions.

### Experimental protocol

We collected measurements while subjects viewed the two scenes in Figure 2 in two different ways: directly and as a projected photograph.

We recruited 38 participants (11 females and 27 males, average age 24.8, SD = 3.1).All 38 participated in the direct viewing experiments. Twelve also participated in the photograph viewing experiment. All subjects were students or staff at the Hong Kong University of Science and Technology. They were all physically healthy with normal or corrected-to-normal visual acuity. The research protocols of our study were approved by the Committee on Research Practices (CRP) of the Hong Kong University of Science and Technology. All subjects provided written consent for their participation.

In the direct viewing experiment, subjects viewed the two designs in two different sessions separated by about 8 weeks. Since the two designs shared same physical space,the time period between the two sessions was required to allow for the construction of the Japanese Garden. Thus, all subjects were first exposed to the simple courtyard, and then to the Japanese garden. 

At the start of each session, subjects entered a white tent, which was closed as the subjects entered so that they could not see the scene. After seating the subject, the experimenter described the experimental procedures. The subject was told that they would be viewing a scene and were free to view it as they liked. There was no other task assigned to the subjects. The experimenter then set up the measurement sensors and calibrated the eye tracker by asking the subject to look at a sequence of five points on a planar screen.Physiological signals and eye gaze werecollected for an initial three-minute baseline while the tent remained closed.The curtain of the tent was then opened, revealing one of the scenes in Figure 2, which the subject viewed freely.After fifteen minutes of data collection, the experimenter collected extra eye tracker calibration data required for aligning the scene camera and eye tracker coordinates. The subject was asked to lookat eight points in the environment, which were identified using a laser pointer.

The photograph viewing experiment followed a similar protocol as the direct viewing experiments, except that subjects were exposed to projected photographs of the two landscapes in one session.The scene order was randomized for each subject. A 15-minute break separated the viewing of the two photographs. Collection of gaze and physiological measurementswereidentical to that in the direct viewing sessions: three minutes of baseline measurements taken as the subject viewed a blank wall followed by 15 minutes of measurements with the photograph projected onto the wall.

### Gaze analysis

Eye gaze trajectories while humans view a scene can be described in many ways. Here we used fixation duration, saccade amplitude, spatial entropy, and transition entropy. 

To compute fixation duration and saccade amplitude, we used the IHMM to separate the gaze trajectory into fixations and saccades(21).

We computed spatial entropy from the gaze heatmap. Denoting the eye gaze at time *t* by $g_{t}\in R^{2}$ and indexing image pixels by $x\in Z^{2}$ , the heat map is calculated by

**(1):**
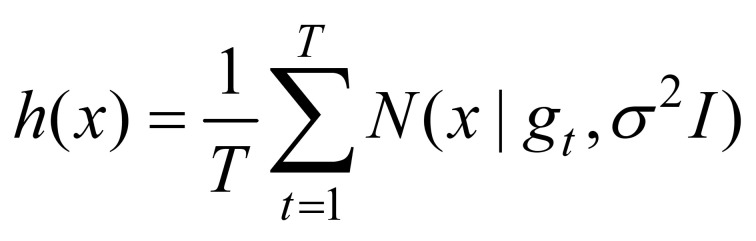

where σ=10 pixels. The spatial entropy is given by 

**(2):**
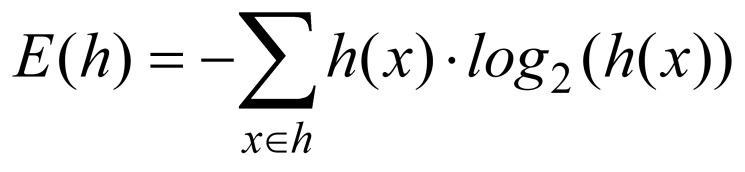

Average fixation duration is often used as a measure of focus during visual behavior(22, 23). The saccade amplitude and spatial entropy are both used to characterize the degree of spatial exploration (23, 24). Larger saccade amplitude and larger spatial entropy both suggest more spatial exploration. 

The three measures above capture either the spatial or the temporal aspects of the gaze trajectory, but not its joint spatio-temporal characteristics. For this, we use the gaze transition entropy (25), which captures the statistics of the transitions between areas of interest in a scene. Lower transition entropies are associated with greater curiosity and more deliberative scanning (26).

The gaze transition entropy is calculated from the parameters of a hidden Markov model (HMM) of the gaze trajectory (27), which we describe in more detail below. 

We denote the gaze location in the image of the scene camera at time *t* by $g_{t}\in R^{2}$ .The HMM assumes that the gaze at time *t* is generated by a discrete state $s_{t}$ ,and that the sequence of states is a Markov chain. For reasons detailed below, we assume two states, i.e. $s_{t}\in \{1,2\}$.

At each time *t*, the emission density is represented by astate-dependent Gaussian mixture model (GMM),

**(3):**
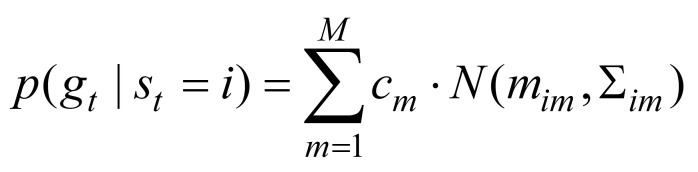

where i∈{1,2} and *M* is the number of mixtures. N(m,Σ) is a 2D Gaussian with mean *m* and covariance matrix Σ. The $c_{\mathrm{im}}$ parameters are mixing parameters that sum to one over *m* for each *i* .

Transitions between states over time are governed by a state transition matrix ${\left[a_{ij}\right]}_{i,j=1}^{2}$ , where $a_{ij}=p(s_{t}=j|s_{t-1}=i)$ . The initial state probabilities at time 0 are given by $p_{i}=p(s_{0}=i)$ . Eventually, the state probabilities settle to a steady state distribution ${\left[\pi_{i}\right]}_{i=1}^{2}$ , which is the eigenvector of the transition probability matrix corresponding to the eigenvalue zero.

The transition entropy is defined by

**(4):**
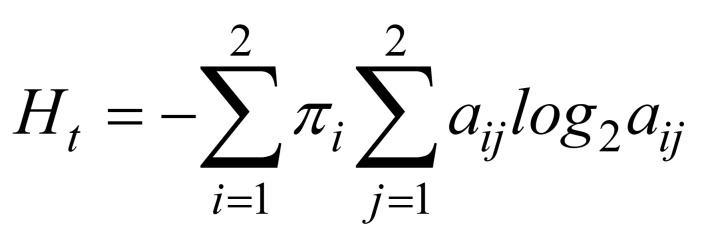

It achieves its maximum (1 bit)when the transition probabilities are uniform (rapid transitions in gaze), and its minimum (0 bits) if for all *i* ,$a_{ij}$ equals one for one value of *i* and is zero otherwise (no transitions).

Based on the measured eye gaze, we found that subjects mainly looked at the center of the visual environment but made occasional saccades to the periphery.Hence, we used two-state HMMs to model gaze trajectories. One state (the central state) modeled the gaze in the central area. The other (the peripheral state) modeled gaze distributed over the periphery.

Since there were few visual elements in the simple courtyard scene, we used four Gaussian mixtures to represent the central state and nine Gaussian mixtures to represent the peripheral state. For the Japanese garden, we used three mixtures to represent the central state and twelve mixtures to represent the peripheral state.

We first trained subject independent HMMs for each environment by pooling the data of all subjects. The means and covariances of the GMMs were initialized manually, based on visual inspection of the measured heat maps.The other HMM parameters (GMM mixture weights, transition probabilities, and initial state probabilities) were all initialized randomly.

For each subject and each environment, we then estimated the transition entropy during each of the five disjoint three minute intervals making up the 15-minute observation period by re-estimating the transition probabilities from the data in each three-minute period while keepingthe GMM and prior state probabilitiesfixed.

### Physiological signal analysis

Unlike previous research which considered only change in pulse rate (9, 10, 12), we considered changes in heart rate, heart rate variability and skin conductance level as measures of autonomic neural system activity.

We used the Pan-Tompkins algorithm to localize heart beats temporally in the electrocardiogram data (28). We used the average heart rate (HR) and the root mean square of successive differences (RMSSD), a measure of heart rate variability, within each three-minute interval as indexes of stress (29).Heart rate is often used as a measure of stress relaxation. Increases in heart rate suggest more stress. Decreases suggest more relaxation.The short-term heart rate variability is not affected by the change of breathing pattern (30). Increases in heart rate variability indicate more relaxation (less stress)(31).

From the galvanic skin response data, we computed the mean skin conductance level (SCL)(32). Decreases in SCL indicate more relaxation. 

Measurements were all taken relative to the averages in the three-minute baseline period. These differences are denoted by ΔHR ,ΔRMSSD ,ΔSCL .

## Results

The average calibration accuracy over all subjects was is about 0.79 degrees. We did not find any significant group differences between females and males on accuracy (p value = 0.96). Gaze measurements when subjects looked away or blinked were discarded (20% of the data on average). We did not find any significant group differences between females and males on data loss (p value = 0.58).

### Gaze behavior under direct viewing

**Figure 3.**
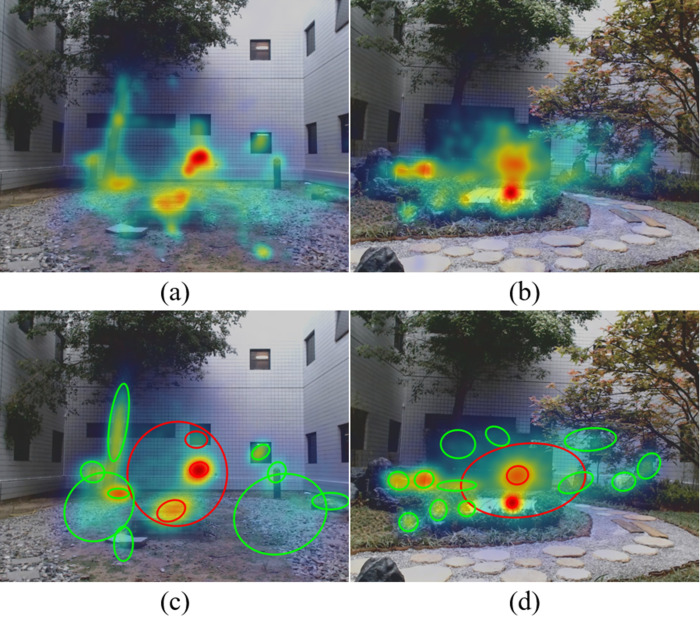
The first row shows measured gaze heat maps in the (a) simple courtyard and (b) Japanese garden. Red/blue indicates more/less eye gaze. The second row shows the heat maps generated by the HMMs for the (c) simple courtyard and (d) Japanese garden, and the covariance ellipses of the GMM states. Red/green ellipses correspond to the central/peripheral states.

Figures3 (a, b) show the average heat maps of the measured gaze trajectories across all participants. Figures 3 (c, d) shows the covariance ellipses of the GMMs, and heat maps of gaze trajectories generated by the HMMs. We found that the Kullback–Leibler Divergence between the HMM generated heat map and the measured heat map to be small relative to the entropy of the measured heat maps (0.07 bits vs 18.32 bits for the simple courtyard and 0.13 bits vs 18.19 bits for the Japanese garden), suggesting that the two state HMM describes the gaze behavior quite well.

Table 1 shows the results of a 2 × 2 (Scene × Gender) ANOVA for the gaze measures. Significance was established at 0.05, corresponding to the 95% confidence level. We used Partial Eta Squared$\eta_{p}^{2}$ to calculate the effect size here, with 0.01, 0.06, and 0.14 corresponding to small, medium, and large effect sizes. We did not observe any significant interaction effect between scene and gender for any of the gaze metrics.Table 2summarizes the statistics of the gaze measures.

**Table 1:** Results of the 2 × 2 (Scene × Gender) ANOVA for gaze measures

	Scene (S)		Gender (G)		S×G	
	F	p	F	p	F	p
Saccade Amplitude	0.41	0.52	**8.89**	**< 0.005**	0.41	0.52
Spatial Entropy	0.01	0.93	**16.5**	**< 0.005**	2.48	0.12
Fixation Duration	2.35	0.13	1.6	0.21	0.47	0.49
Transition Entropy	**190**	**< 0.005**	**14.6**	**< 0.005**	0	0.96

**Table 2:** Gaze statistics (mean ± standard deviation) when viewing the two scenes directly. (SC: simple courtyard, JG: Japanese Garden; M: Male, F: female, All: all subjects.

		Saccade Amplitude	Spatial Entropy	Fixation Duration	Transition Entropy
SC	M	106±26	16.3±0.5	345±152	0.58±0.18
	F	119±35	16.5±0.6	328±95	0.66±0.13
	All	110±30	16.3±0.6	341±133	0.60±0.17
JG	M	106±31	16.1±1.1	422±213	0.31±0.17
	F	114±35	16.6±0.4	355±123	0.38±0.16
	All	109±32	16.3±1.0	406±198	0.34±0.17

We observed a significant effect for scene only with the transition entropy measure (*p*< 0.005, effect size = 0.38), which was lower in the Japanese garden than in the simple courtyard. As discussed earlier, lower transition entropy is associated with more curiosity and more deliberative scanning. Thus, our results suggest that subjects were more visually engaged when viewing the Japanese garden, but that only the measure that takes into account the short term spatio-temporal statistics of the gaze can detect this difference.

**Figure 4.**
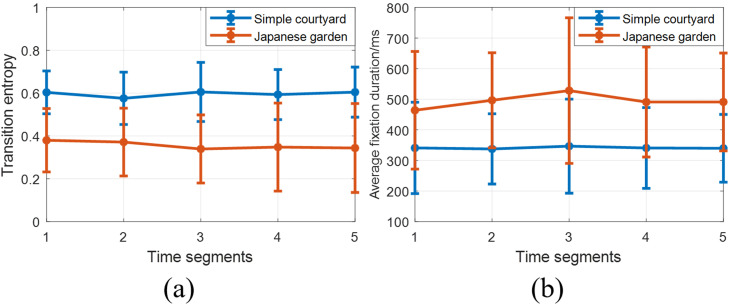
Plots of transition entropy (a) and fixation duration (b) averaged across all subjects versus time in simple courtyard and the Japanese garden. Error bars indicate standard deviation.

Over longer time intervals, gaze behavior did not vary. Figure 4 shows that transition entropy (a) and fixation duration (b) in the two environments both remained constant over time. We observed similar curves for the other gaze measures (data not shown).

We observed significant effects of gender for saccade amplitude (*p*< 0.005, effect size = 0.04), spatial entropy (*p*< 0.005, effect size = 0.03), and transition entropy (*p*< 0.005, effect size = 0.15), but not for fixation duration. Females had larger average saccade amplitude, larger spatial entropy, and larger transition entropy than males. Transition entropy had the largest effect size. These results suggest that female subjects tended to be less focused and engage in more spatial exploration than males when viewing the gardens.

To further elucidate gender differences in gaze behavior, we modelled the distributions of gaze points using Gaussian Mixture Models (GMMs), which allowed us to identify regions of interest in the environment automatically and to measure the relative proportion of time subjects spent viewing them. Each Gaussian density models a region of interest. The entire gaze history is modelled as a weighted mixture of these Gaussian densities. 

In each environment, we selected the number of mixtures that minimized the Bayesian information criterion (BIC). This criterion avoids over-fitting resulting from too many parameters by adding a term penalizing the model complexity. We refer readers to (33)for more details on GMMs and model comparison. The number of Gaussian mixtures minimizing the BIC was 13 for simple courtyard and 14 for the Japanese garden.

To identify the GMM parameters (mixture means, covariances and weights), we first fit one GMM for each environment using the data from all subjects (both male and female). This enabled us to identify common regions of interest for males and females. In the second step, we fixed the mixture means and covariances and re-estimated two sets of mixture weights: one for females and one fore males only. Differences in the mixture weights between genders reflect different levels of interest in the same image regions.

**Figure 5.**
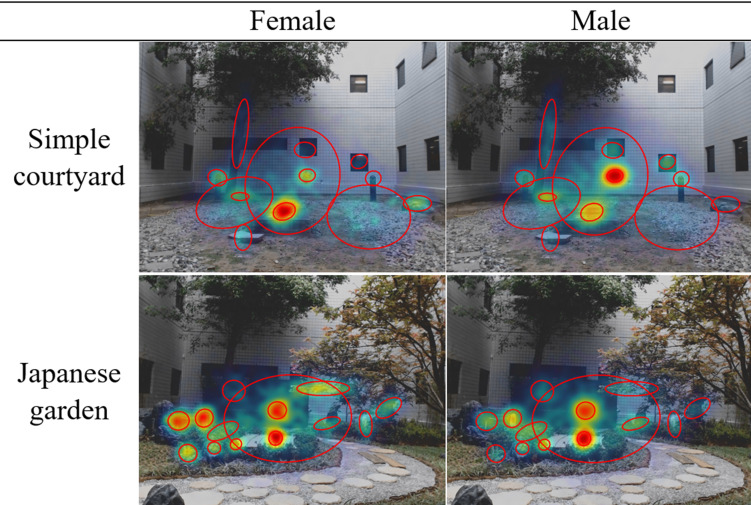
The plots show the heat maps of the gaze trajectories generated by Gaussian mixture models with the same mixture means and covariances, but different mixture weights. Red indicatesmore frequently fixated areas. Blue indicates less frequently fixated areas. The ellipses are the covariance ellipses of the Gaussian clusters.

Figure 5 compares gaze heat maps generated by the female and male GMMs.When viewing the simple courtyard, females focus more on the central concrete block, whereas males focus more on the water hose attached to the wall. In the Japanese garden, females and males both spend considerable amounts of time gazing at the tatami mat covering the concrete block and at the bench in the center of the scene. However, females spend a larger proportion of time also gazing at points in the periphery, both in the simple courtyard and in the Japanese garden, leading to more spatially distributed gaze maps.

To quantify the spatial spread of the gaze maps, we calculate the determinant of the weighted between cluster covariance matrix:

**(5):**
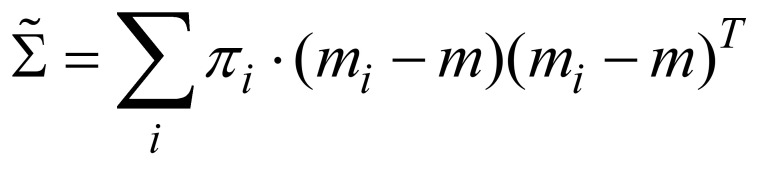

where $m=\sum_{i}\pi_{i}m_{i}$ is the weighted mean of clusters. Larger determinants correspond to larger spatial spread. For both the simple courtyard and the Japanese garden, the determinants of the weighted between-cluster covariance for females (9.2395e+07 and 1.5819e+07) is larger than that for males (7.8258e+07 and 8.1833e+06). This finding is consistent with our earlier results suggesting that males were more focused than femalesin both two environments.

### Physiological responses under direct viewing

Table 3 shows the results of a 2 × 2 (Scene × Gender) ANOVA for the physiological responses in the final three-minute viewing segment. We did not observe any significant interaction effect between scene and gender for any of the physiological responses. 

**Table 3:** Results of the 2 × 2 (Scene × Gender) ANOVA for physiological measures

	Scene (S)		Gender (G)		S×G	
	F	p	F	p	F	p
ΔHR	**3.86**	**0.05**	**4.04**	**0.05**	0	0.94
ΔRMSSD	0.10	0.76	0.98	0.33	0.26	0.61
ΔSCL	1.76	0.19	3.29	0.07	1.39	0.24

**Figure 6.**
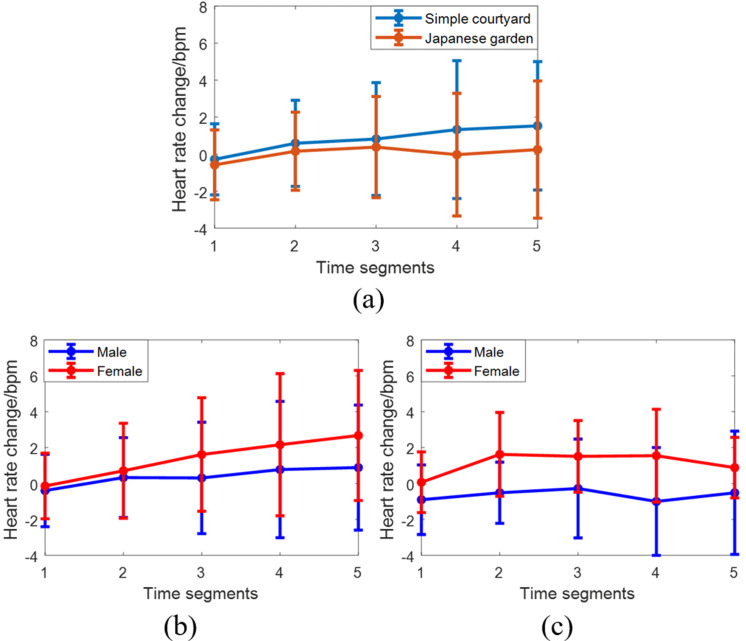
(a) Plots of theaverage ΔHR across all subjects versus time in the simple courtyard (blue) and the Japanese garden (red). (b,c) Comparison of the average ΔHR's across female (red) and across males (blue) versus time in (b) the simple courtyard and (c) the Japanese garden. Error bars indicate standard deviation.

We observed a significant effect for scene for ΔHR (*p* = 0.05, effect size = 0.06), but not for ΔRMSSD and ΔSCL as shown in Table 3. Figure 6(a) plots the ΔHR averaged across all subjects versus time (in three minute segments) in the simple courtyard and the Japanese garden. The ΔHR increases when viewing the simple courtyard but remains relatively constant when viewing Japanese garden. Our ANOVA analysis indicates that the final ΔHR is significantly lower in the Japanese garden than in the simple courtyard.

We observed a significant effect of gender for ΔHR (*p* = 0.05, effect size = 0.06), but not for ΔRMSSD and ΔSCL . Figure 6(b) compares the average ΔHR over time for female and male subjects in the simple courtyard. The average heart rate of the female subjects increased steadily over the viewing period, whereas the average heart rate of the male subjects remained relatively constant over time. Figure 6(c) compares the average ΔHR in the Japanese garden. When viewing the Japanese garden, the average heart rates of both female and male subjects remained relatively constant over time. However, consistent with the results in the simple courtyard, the ΔHR of female subjects was higher than for male subjects.Our results suggest that female subjects felt more stress in both environments. 

### Interaction between gaze behavior and physiological responses

**Figure 7.**
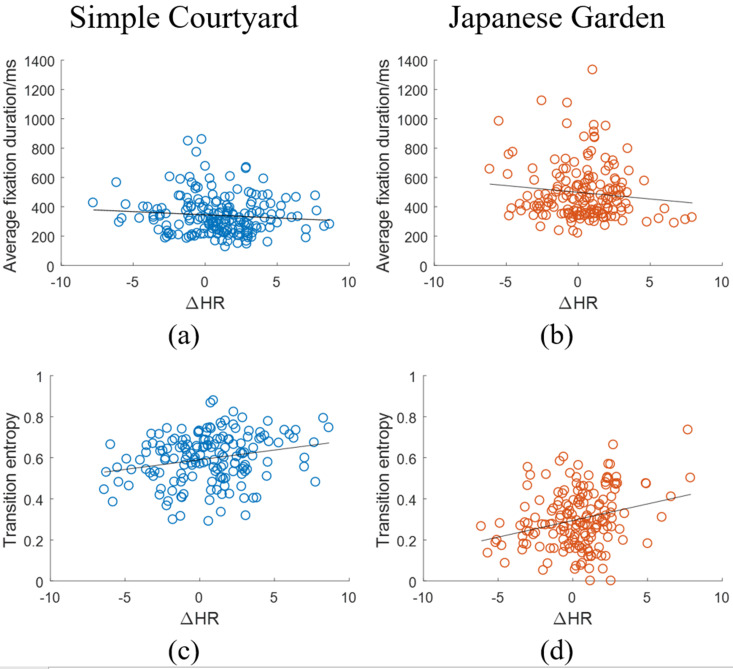
Scatter plots of the average fixation duration (a,b)(top row) and the transition entropy (c,d)(bottom row) versus the ΔHR for the simple courtyard (a,c)(left column)and the Japanese garden (b,d)(right column).Each dot represents the data calculated from one 3-minute interval of viewing.

Figure 7 shows scatter plots of average fixation duration (a,b) and transition entropy (c,d) versus ΔHR for all participants when viewing the simple courtyard (a,c) and the Japanese garden (b,d). We did not find a significant correlation between average fixation duration and ΔHR in either environment (first row in Table 4). However, we did find a small, but statistically significant positive correlation between the transition entropy and ΔHR in both the simple courtyard (0.22, p = 0.005) and the Japanese garden (0.27, p< 0.005)(first row in Table 5). 

**Table 4:** Correlations with fixation duration

CC/p value	Simple courtyard		Japanese Garden	
	CC	p	CC	p
ΔHR	-0.09	0.21	-0.11	0.14
ΔRMSSD	0.02	0.83	0.08	0.23
ΔSCL	-0.03	0.72	-0.02	0.85

**Table 5:** Correlations with transition entropy

CC/p value	Simple courtyard		Japanese Garden	
	CC	p	CC	p
ΔHR	**0.22**	**0.005**	**0.27**	**< 0.005**
ΔRMSSD	-0.03	0.71	0.05	0.48
ΔSCL	0.04	0.61	0.02	0.77

**Table 6:** Correlations with saccade amplitude

CC/p value	Simple courtyard		Japanese Garden	
	CC	p	CC	p
ΔHR	0.12	0.09	0.12	0.10
ΔRMSSD	0.05	0.50	0.07	0.37
ΔSCL	-0.02	0.80	0.06	0.40

**Table 7:** Correlations with spatial entropy

CC/p value	Simple courtyard		Japanese Garden	
	CC	p	CC	p
ΔHR	-0.02	0.83	0.12	0.11
ΔRMSSD	0.09	0.20	0.05	0.50
ΔSCL	0.00	0.99	-0.07	0.32

The positive correlation between transition entropy and ΔHR was not affected by the number of mixtures used in the GMM observation probabilities of the HMM models. We varied the number of mixtures times by factors of 1.25 and 0.75 for the two environments (16 mixtures and 10 mixtures for the simple courtyard, 19 mixtures and 11mixtures for the Japanese garden). In all cases, the correlation coefficient remained in the same range, and retained statistical significance. In the simple courtyard, the correlation coefficients were 0.23 (*p*< 0.005) for 16 mixtures and 0.21 (*p* = 0.007). In the Japanese garden, they were 0.28 (*p*< 0.005)for 19 mixtures and 0.29 (*p*< 0.005) for 11 mixtures.

We found no statistically significant correlations between the gaze measures and the other physiological measures (Tables 4-7).

### Effect of presentation modality

Table 8 summarizes the gaze measure statistics. Table 9 shows the results of a 2 × 2 (Scene × Modality) ANOVA for gaze measures.We calculated the effect size using Partial Eta Squared $\eta_{p}^{2}$ . We found no significant interaction effects between scene and modality for any gaze metrics, except for transition entropy (p < 0.005).

**Table 8:** Gaze measures for two viewing modalities of the two environments.

	Simple Courtyard		Japanese Garden	
	Direct	Photo	Direct	Photo
Saccade Amplitude	108±33	142±53	121±40	149±55
Spatial Entropy	16.6±0.4	16.9±0.5	16.6±0.4	16.9±0.5
Fixation Duration	322±107	326±126	382±124	342±134
Transition Entropy	0.59±0.19	0.43±0.22	0.40±0.18	0.46±0.19

**Table 9:** Results of the 2 × 2 (Stimuli × Modality) ANOVA for gaze measures.

	Scene (S)		Modality (M)		S×M	
	F	p	F	p	F	p
Saccade Amplitude	2.63	0.52	**27.45**	**< 0.005**	0.25	0.62
Spatial Entropy	0.05	0.82	**18.7**	**< 0.005**	0.09	0.76
Fixation Duration	4.08	0.05	0.9	0.34	1.32	0.25
Transition Entropy	**7.41**	**0.007**	**4.9**	**0.03**	**13.3**	**<0.005**

Consistent with our previous results with direct viewing only, we found no significant effect of scene for saccade amplitude, spatial entropy and fixation duration. However, we did find significant effect of modality for the spatial measures (saccade amplitude and spatial entropy), but not the temporal measure (fixation duration). Average saccade amplitude increased when subjects viewed the projected photographs compared towhen they viewed the spaces directly (*p*< 0.005, effect size = 0.10). Spatial entropy also increased when viewing the projected photograph versus when viewing directly (*p*< 0.005, effect size = 0.11).

**Figure 8.**
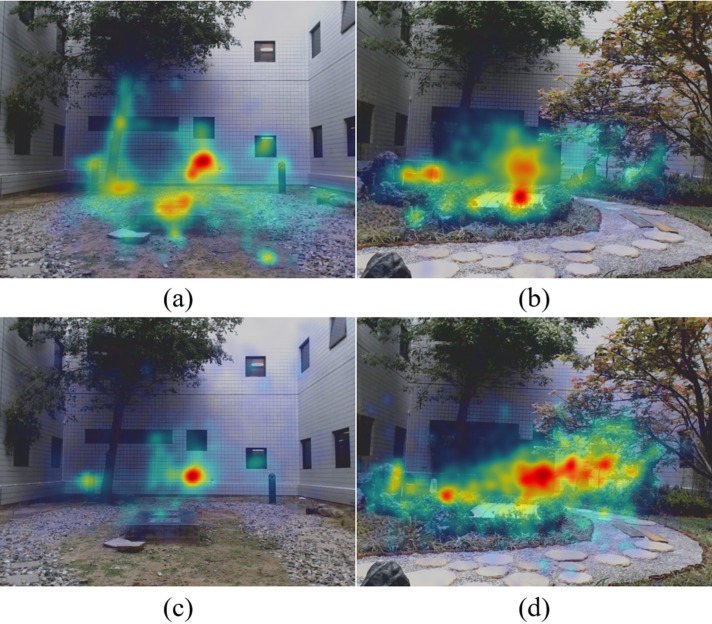
The first row shows measured gaze heat maps when viewing directly (a) the simple courtyardand (b) the Japanese garden. The secondrow shows measured gaze heat mapswhen viewing projected photographs of (c) the simple courtyard and (d) the Japanese garden viewing. Red/blue indicates more/lessfrequent eye gaze.

These results suggest that gaze was more spatially spread when subjects viewed the photograph than when they viewed directly. This is consistent with the gaze heat maps shown in Figure 8. In the simple courtyard, we see more frequent excursions to the upper part of the environment (e.g. the second floor windows). In the Japanese garden, we observe the gaze is generally more broadly spread, and there are also frequent excursions to the upper part of the environment.

As suggested by the significant interaction effect, the effect of scene and modality on transition entropy is more complex. For the simple courtyard, the transition entropy when viewing the projected photograph is smaller than when viewing directly. However, for the Japanese garden, the transition entropy when viewing the projected photograph is larger.

Table 10 shows the results of a 2 × 2(Scene × Modality) ANOVA for the physiological measures in the final three-minute viewing segment. We did not observe any significant effect for scene, modality or interaction.

**Table 10:** Results of the 2 × 2 (Scene × Modality) ANOVA for physiological measures

	Scene (S)		Modality (M)		S×M	
	F	p	F	p	F	p
ΔHR	0	0.95	1.21	0.28	0.68	0.41
ΔRMSSD	0.84	0.37	2.94	0.09	2.15	0.15
ΔSCL	0.73	0.40	0.79	0.38	1.61	0.21

## Discussion

This work has described the first set of tightly synchronized measurements of gaze behavior and physiological responses to viewing a Japanese garden. Our analysis has confirmed previous findings about the relative effects of Japanese gardens on parasympathetic nervous system activity. We alsofound an interaction between gaze behavior and physiological responsesand examined the gender differences. In comparison toprevious work, we conducted experiments on a much younger subject group.

In line with previous research, we found that subjects’ physiological responses were consistent with greater parasympathetic nervous system activity (less stress) when they viewedthe Japanese garden than when they viewed the simple courtyard. However, in contrast to previous work (9, 10, 12, 13), we did not find decreases in heart rate upon exposure to the Japanese style garden. There are at least two possible factors which account for this discrepancy. One factor is a difference in familiarity to the garden style, which might lead to different aesthetic preferences. The subjectsin(9) were landscape architecture students. Therefore, they would be both more interested in and more familiar in the garden scene.However, most of our participants were engineering students. Another factor might be the age difference. Our subjects were much younger than those in previous studies. The youth generally have less interest in nature than the elderly (34). They may also be less patient. According to the feedback from subjects, many of them felt uncomfortable sitting still for 15 minutes.

According to (14), Japanese gardens are designed to facilitate meditation by encouraging viewers to slow down their gaze and make fewer gaze transitions. Objects are more distinct so that viewer do not need to search as much. For example, once viewers’ gaze reached the central part of the Japanese garden, there was a Japanese bench for them to rest their gaze on, as well as numerous auxiliary elements via which they could visually approach the bench.

Consistent with these design principles, we found that subjects exhibited more focused gaze behavior in the Japanese garden. The lower transition entropy in the Japanese garden compared to the simple courtyard indicate that subjects made less frequent transitions between the center and the periphery of the scene. Compared with the simple courtyard, the Japanese garden has more visual elements, which can lead the subject more slowly and indirectly towards the center.While the simple courtyard also has elements in the center (the concrete block and the water hose), when the eyes reach the center of the scene, there are fewer auxiliary elements to focus on. As a result, subjects’ gaze transitions rapidly between the center and periphery.However, there are also other potential explanations for our observations. Looking at a simple courtyard for fifteen minutes may be very boring for the subject. There may be some systematic effects due to unbalanced presentation order, Due to the constraints involved in using the same physical space for both environments, all subjects were first exposed to the simple courtyard, and then to the Japanese garden.

Consistent with the design goal of to slowing down gaze to encouraging meditation, we found that more focused gaze behavior (lower transition entropy) had a weak positive correlation with greater relaxation (decrease in mean heart rate change).

Our results on gender lend further support to many prior experimental findings on the gender differences of gaze behaviors, which were conducted inother stimulus and experimental settings (18, 19, 20). This past work has found that females follow a more exploratory viewing strategy than males. We also found that the gaze behavior of femaleswas significantly different than that of males,e.g. larger saccade amplitude, larger spatial entropy and larger transition entropy. These are all consistent with less focus and more exploration. Furthermore, we characterized gender differences in gaze heat maps using Gaussian mixture models. Our results suggest that the females’ attention has distributed more evenly among the different elements than the males’ attention. 

The observed gender differences in gaze behavior are consistent with the observed gender differences in physiological responses. Our correlational analysis suggests that on short time scales (3 minutes), more focused gaze behavior (as measured by lower transition entropy) is correlated with greater parasympathetic nervous system activity (as measured by lower heart rate). Similarly, across gender, we find that males exhibit more focused gaze behavior and greater parasympathetic nervous system activity than females. 

Many studies have suggested that photographs can be treated as valid surrogates for on-site viewing (35, 36, 37). However, other studies suggest that the experience of viewing a scene directly differs significantly from the experience of viewing a photograph of that scene (38, 39, 40). Goto et al.compared viewing a garden directly with viewing it through a window, and found that subjects paid more attention to plants and had stronger responseswhen viewing directly (13). 

Our results do not support the hypothesis that photographs can be treated as valid surrogates for on-site viewing, as they do not elicit the same gaze behavior. We found that when subjects viewedprojected photographs of the scenes, their gaze trajectorieswere more widely distributed spatially than when they viewed directly. We speculate that this is due to the 3D cues available when viewing directly, which may provide an additional cue to help focus viewers’ attention on the garden structures. In contrast, when viewing a projected photograph, viewers can only rely on intensity, texture and color cues. 

### Conclusion

The system we have developed enables simultaneous and synchronized measure of gaze behavior and physiological measure as subjects view a wide range of different environments, while causing little to no discomfort or stress to the subject. The use of this system may lead to new studies and insights into how gaze behavior evoked by different landscape designs may lead to different feelings in the user. The system is applicable to a much wider range of settings than considered here. For example, it may be used in studies of people’s responses to indoor environments. Many people spend the vast majority of their time indoors. Thus, the indoor environment can have a profound impact on health. Proper design of living environments, both indoor and outdoor, holds great promise as non-pharmacological therapy to improve well-being.

One of the key benefits of our system is that it enables us to study the interaction between the environment, a person’s visual behavior in that environment, and that person’s physiological responses. Our approach recognized that perception, even by a voluntary observer in a static environment, is dynamic and active.In our experiment, even though subjects were seated while observing the environments, there were not passive.Rather, they consciously and unconsciously directed their attention towards different scene elements over time.Many of these attention shifts are accompanied by saccadic eye movements, which direct eye gaze so that the fovea falls upon different objects of interest in the environment.Thus, gaze behavior is a spatial-temporal process that not only reflects, but may also influence, subjects internal responses to viewing a scene.

Our work has revealed evidence of this interaction. In particular, we find that the gaze transition entropy, a measure that depends upon the spatio-temporal characteristics of the gaze trajectory, is positively correlated with changes in heart rate. Since decreased transition entropy is associated with increased focus or engagement and decreased heart rate is associated with increased relaxation, this suggests that more focused gaze behavior is connected with more relaxation.

We have also studied differences due to scene content, presentation modality and gender, and found significant effects along all these dimensions. Subjects were more focused and relaxed in the Japanese garden compared with the control. Gaze was more concentrated spatially when subjects viewed a scene directly rather than as a photograph. Females exhibited less focused and more exploratory gaze behavior than males, and consistent with our found correlation between relaxation and gaze focus, were less relaxed.

Our initial experiment was performed on healthy young participants. In the future, we will test the responses of elderly persons with advanced dementia. In a pilot test on elderly individuals, our system was well tolerated.We will further compare the effect of different viewing modalities, e.g. direct, as a photograph and in virtual modality.

### Ethics and Conflict of Interest

The author(s) declare(s) that the contents of the article are in agreement with the ethics described in http://biblio.unibe.ch/portale/elibrary/BOP/jemr/ethics.html and that there is no conflict of interest regarding the publication of this paper. 

### Acknowledgements

This work was supported by the Hong Kong University of Science and Technology, through grant number IEG16SC01.

## References

[b33] Bishop CM Pattern Recognition and Machine Learning. 2006.

[b27] Chuk,T. , Chan,A. B. , & Hsiao,J. H. (2014).Understanding eye movements in face recognition using hidden Markov models. Journal of Vision (Charlottesville, Va.),14(11),8. 10.1167/14.11.8 1534-7362 25228627

[b25] Ciuperca,G. , & Girardin,V. (Eds.) On the estimation of the entropy rate of finite Markov chains. Proceedings of the International Symposium on Applied Stochastic Models and Data Analysis;2005.

[b18] Coutrot,A. , Binetti,N. , Harrison,C. , Mareschal,I. , & Johnston,A. (2016).Face exploration dynamics differentiate men and women. Journal of Vision (Charlottesville, Va.),16(14),16. 10.1167/16.14.16 1534-7362 27893894

[b35] Daniel,T. C. (1976).Criteria for development and application of perceived environmental quality indices. Perceiving Environmental Quality.Springer.

[b34] Dunnett,N. , & Qasim,M. (2000).Perceived benefits to human well-being of urban gardens. HortTechnology,10(1),40–45. 10.21273/HORTTECH.10.1.40 1063-0198

[b11] Elsadek,M. , Sun,M. , Sugiyama,R. , & Fujii,E. (2019).Cross-cultural comparison of physiological and psychological responses to different garden styles. Urban Forestry & Urban Greening,38,74–83. 10.1016/j.ufug.2018.11.007 1618-8667

[b3] Friedrich,M. J. (2009).Therapeutic environmental design aims to help patients with Alzheimer disease. Journal of the American Medical Association,301(23),2430. 10.1001/jama.2009.809 0098-7484 19531775

[b23] Gameiro,R. , Kaspar,K. , König,S. U. , Nordholt,S. , & König,P. (2017).Exploration and Exploitation in Natural Viewing Behavior. Scientific Reports,7(1),2311. 10.1038/s41598-017-02526-1 2045-2322 28536434 PMC5442137

[b38] Gibson,J. J. (2014).The ecological approach to visual perception: classic edition.Psychology Press 10.4324/9781315740218

[b9] Goto,S. , Kamal,N. , Puzio,H. , Fujii,E. , & Herrup,K. (2013).Psychological and Biological Response to Three Landscapes in Japan: A Pilot Study. Journal of Therapeutic Horticulture.,23(1).

[b13] Goto,S. , Gianfagia,T. J. , Munafo,J. P. , Fujii,E. , Shen,X. , Sun,M. , Shi,B. E. , Liu,C. , Hamano,H. , & Herrup,K. (2017).The power of traditional design techniques: The effects of viewing a Japanese garden on individuals with cognitive impairment. HERD: Health Environments Research & Design Journal.,10(4),74–86. 10.1177/1937586716680064 2167-5112 28643564

[b12] Goto,S. , Kamal,N. , Puzio,H. , Kobylarz,F. , & Herrup,K. (2014).Differential responses of individuals with late-stage dementia to two novel environments: A multimedia room and an interior garden. Journal of Alzheimer’s Disease,42(3),985–998. 10.3233/JAD-131379 1387-2877 25024307

[b14] Goto,S. , & Naka,T. (2015).Japanese gardens: Symbolism and design.Routledge 10.4324/9781315685267

[b10] Goto,S. , Park,B.-J. , Tsunetsugu,Y. , Herrup,K. , & Miyazaki,Y. (2013).The effect of garden designs on mood and heart output in older adults residing in an assisted living facility. HERD: Health Environments Research & Design Journal.,6(2),27–42. 10.1177/193758671300600204 1937-5867 23532694

[b40] Hart,M. B. , Vockeroth,J. , Schumann,F. , Bartl,K. , Schneider,E. , Koenig,P. ,. . . (2009).Gaze allocation in natural stimuli: Comparing free exploration to head-fixed viewing conditions. Visual Cognition,17(6-7),1132–1158. 10.1080/13506280902812304 1350-6285

[b8] Hibi,S. , & Earle,J. (2000).Infinite Spaces; Art and Wisdom of the Japanese Garden, The.Tuttle Publishing.

[b32] Jacobs,S. C. , Friedman,R. , Parker,J. D. , Tofler,G. H. , Jimenez,A. H. , Muller,J. E. , Benson,H. , & Stone,P. H. (1994).Use of skin conductance changes during mental stress testing as an index of autonomic arousal in cardiovascular research. American Heart Journal,128(6 Pt 1),1170–1177. 10.1016/0002-8703(94)90748-X 0002-8703 7985598

[b6] Kaplan R , Kaplan S The experience of nature: A psychological perspective: CUP Archive; 1989.

[b1] Kong,E. H. , Evans,L. K. , Guevara,J. P. , & the Marcus & Barnes (2009).Nonpharmacological intervention for agitation in dementia: A systematic review and meta-analysis. Aging & Mental Health,13(4),512– 520. 10.1080/13607860902774394 1360-7863 19629775

[b26] Krejtz,K. , Duchowski,A. , Szmidt,T. , Krejtz,I. , González Perilli,F. , Pires,A. , Vilaro,A. , & Villalobos,N. (2015).Gaze transition entropy. [TAP] ACM Transactions on Applied Perception,13(1),1–20. 10.1145/2834121 1544-3558

[b15] Liu,C. , Herrup,K. , & Shi,B. E. (Eds.) Remote gaze tracking system for 3D environments. 2017 39th Annual International Conference of the IEEE Engineering in Medicine and Biology Society (EMBC); 2017: IEEE. 10.1109/EMBC.2017.8037186 29060230

[b16] Liu,C. , Zhang,Y. , Herrup,E. K. , & Shi,B. E. (Eds.) On the Interaction between Gaze Behavior and Physiological Responses when Viewing Garden Scenes. 2018 40th Annual International Conference of the IEEE Engineering in Medicine and Biology Society (EMBC); 2018: IEEE. 10.1109/EMBC.2018.8512227 30440401

[b17] Liu,C. , Zhang,Y. , Goto,S. , Sun,M. , Herrup,K. , & Shi,B. (2018).QUANTIFYING INTERACTIONS BETWEEN GAZE BEHAVIOR AND PHYSIOLOGICAL RESPONSES WHEN VIEWING JAPANESE STYLE GARDENS. Alzheimer’s & Dementia,14(7),1508 10.1016/j.jalz.2018.06.2586 1552-5260

[b2] Marcus,C. C. , & Barnes,M. (1999).Healing gardens: Therapeutic benefits and design recommendations.John Wiley & Sons.

[b19] Mercer Moss,F. J. , Baddeley,R. , & Canagarajah,N. (2012).Eye movements to natural images as a function of sex and personality. PLoS One,7(11),e47870. 10.1371/journal.pone.0047870 1932-6203 23248740 PMC3511485

[b24] Mills,M. , Hollingworth,A. , Van der Stigchel,S. , Hoffman,L. , & Dodd,M. D. (2011).Examining the influence of task set on eye movements and fixations. Journal of Vision (Charlottesville, Va.),11(8),17. 10.1167/11.8.17 1534-7362 21799023 PMC3163592

[b29] Moyle,W. , Cooke,M. L. , Beattie,E. , Shum,D. H. , O’Dwyer,S. T. , Barrett,S. , & Sung,B. (2014).Foot massage and physiological stress in people with dementia: A randomized controlled trial. Journal of Alternative and Complementary Medicine (New York, N.Y.),20(4),305–311. 10.1089/acm.2013.0177 1075-5535 24047244 PMC3994911

[b22] Nyström,M. , & Holmqvist,K. (2010).An adaptive algorithm for fixation, saccade, and glissade detection in eyetracking data. Behavior Research Methods,42(1),188–204. 10.3758/BRM.42.1.188 1554-351X 20160299

[b28] Pan,J. , & Tompkins,W. J. (1985).A real-time QRS detection algorithm. IEEE Transactions on Biomedical Engineering,32(3),230–236. 10.1109/TBME.1985.325532 0018-9294 3997178

[b4] Pasha,S. , & Shepley,M. M. (2013).Research note: Physical activity in pediatric healing gardens. Landscape and Urban Planning,118,53– 58. 10.1016/j.landurbplan.2013.05.005 0169-2046

[b30] Penttilä,J. , Helminen,A. , Jartti,T. , Kuusela,T. , Huikuri,H. V. , Tulppo,M. P. , Coffeng,R. , & Scheinin,H. (2001).Time domain, geometrical and frequency domain analysis of cardiac vagal outflow: Effects of various respiratory patterns. Clinical Physiology and Functional Imaging,21(3),365–376. 10.1046/j.1365-2281.2001.00337.x 1475-0961 11380537

[b5] Reeve,A. , Nieberler-Walker,K. , & Desha,C. (2017).Healing gardens in children’s hospitals: Reflections on benefits, preferences and design from visitors’ books. Urban Forestry & Urban Greening,26,48– 56. 10.1016/j.ufug.2017.05.013 1618-8667

[b21] Salvucci,D. D. , & Goldberg,J. H. (Eds.) Identifying fixations and saccades in eye-tracking protocols. Proceedings of the 2000 Symposium on Eye Tracking Research & Applications;2000 10.1145/355017.355028

[b39] Shuttleworth,S. (1980).The use of photographs as an environment presentation medium in landscape studies. Journal of Environmental Management,11(1),61–76.0301-4797

[b36] Stamps,A. E.,III (1990).Use of photographs to simulate environments: A meta-analysis. Perceptual and Motor Skills,71(3),907–913. 10.2466/pms.1990.71.3.907 0031-5125 21162439

[b37] Stewart,T. R. , Middleton,P. , Downton,M. , & Ely,D. (1984).Judgments of photographs vs. field observations in studies of perception and judgment of the visual environment. Journal of Environmental Psychology,4(4),283–302. 10.1016/S0272-4944(84)80001-8 0272-4944

[b31] Terathongkum,S. , & Pickler,R. H. (2004).Relationships among heart rate variability, hypertension, and relaxation techniques. Journal of Vascular Nursing,22(3),78–82. 10.1016/j.jvn.2004.06.003 1062-0303 15371972

[b7] Ulrich,R. S. , Simons,R. F. , Losito,B. D. , Fiorito,E. , Miles,M. A. , & Zelson,M. (1991).Stress recovery during exposure to natural and urban environments. Journal of Environmental Psychology,11(3),201–230. 10.1016/S0272-4944(05)80184-7 0272-4944

[b20] Vassallo,S. , Cooper,S. L. , & Douglas,J. M. (2009).Visual scanning in the recognition of facial affect: Is there an observer sex difference? Journal of Vision (Charlottesville, Va.),9(3),11.1–10. 10.1167/9.3.11 1534-7362 19757950

